# The suboptimal fibrinolytic response in COVID‐19 is dictated by high PAI‐1

**DOI:** 10.1111/jth.15806

**Published:** 2022-07-21

**Authors:** Claire S. Whyte, Megan Simpson, Gael B. Morrow, Carol A. Wallace, Alexander J. Mentzer, Julian C. Knight, Susan Shapiro, Nicola Curry, Catherine N. Bagot, Henry Watson, Jamie G. Cooper, Nicola J. Mutch

**Affiliations:** ^1^ Aberdeen Cardiovascular & Diabetes Centre, School of Medicine, Medical Sciences and Nutrition, Institute of Medical Sciences University of Aberdeen Aberdeen UK; ^2^ Radcliffe Department of Medicine University of Oxford Oxford UK; ^3^ Oxford Haemophilia & Thrombosis Centre, NIHR Oxford Biomedical Research Centre Oxford University Hospitals NHS Foundation Trust Oxford UK; ^4^ Wellcome Centre for Human Genetics University of Oxford Oxford UK; ^5^ Department of Haematology Glasgow Royal Infirmary Glasgow UK; ^6^ Emergency Department Aberdeen Royal Infirmary, NHS Grampian Aberdeen UK; ^7^ School of Medicine, Medical Sciences and Nutrition University of Aberdeen Aberdeen UK

**Keywords:** COVID‐19, fibrin, fibrinolysis, PAI‐1, vitronectin

## Abstract

**Background:**

Severe COVID‐19 disease is associated with thrombotic complications and extensive fibrin deposition. This study investigates whether the hemostatic complications in COVID‐19 disease arise due to dysregulation of the fibrinolytic system.

**Methods:**

This prospective study analyzed fibrinolytic profiles of 113 patients hospitalized with COVID‐19 disease with 24 patients with non‐COVID‐19 respiratory infection and healthy controls. Antigens were quantified by Ella system or ELISA, clot lysis by turbidimetric assay, and plasminogen activator inhibitor‐1 (PAI‐1)/plasmin activity using chromogenic substrates. Clot structure was visualized by confocal microscopy.

**Results:**

PAI‐1 and its cofactor, vitronectin, are significantly elevated in patients with COVID‐19 disease compared with those with non‐COVID‐19 respiratory infection and healthy control groups. Thrombin activatable fibrinolysis inhibitor and tissue plasminogen activator were elevated in patients with COVID‐19 disease relative to healthy controls. PAI‐1 and tissue plasminogen activator (tPA) were associated with more severe COVID‐19 disease severity. Clots formed from COVID‐19 plasma demonstrate an altered fibrin network, with attenuated fiber length and increased branching. Functional studies reveal that plasmin generation and clot lysis were markedly attenuated in COVID‐19 disease, while PAI‐1 activity was elevated. Clot lysis time significantly correlated with PAI‐1 levels. Stratification of COVID‐19 samples according to PAI‐1 levels reveals significantly faster lysis when using the PAI‐1 resistant (tPA) variant, tenecteplase, over alteplase lysis.

**Conclusion:**

This study shows that the suboptimal fibrinolytic response in COVID‐19 disease is directly attributable to elevated levels of PAI‐1, which attenuate plasmin generation. These data highlight the important prognostic potential of PAI‐1 and the possibility of using pre‐existing drugs, such as tenecteplase, to treat COVID‐19 disease and potentially other respiratory diseases.

**Essentials**

- Severe COVID‐19 disease is associated with thrombosis and fibrin deposition.
- This study investigates whether thrombotic complications arise due to dysregulation of the fibrinolysis.
- Plasminogen activator inhibitor‐1 (PAI‐1) and its stabilizing cofactor, vitronectin, are significantly elevated in patients with COVID‐19 disease.
- Studies with tissue plasminogen activator variants reveal that PAI‐1 drives hypofibrinolysis in COVID‐19 disease.

## 1 INTRODUCTION

Thrombosis is a common feature of severe COVID‐19 disease arising from infection with severe acute respiratory syndrome coronavirus 2 (SARS‐CoV‐2).^1^, ^2^ Venous thromboembolism, particularly pulmonary embolism (PE),^3^, ^4^ but also arterial thromboembolic complications, are also prevalent.^5^ Large vessel thrombi are present in almost half of patients who are critically ill with COVID‐19 and focal platelet and fibrin microthrombi are described in over 80% of cases.^6^ Importantly, these thromboembolic complications are observed despite prophylactic and full dose anticoagulation.^4^

The coagulopathy seen in COVID‐19 disease is characterized by elevated fibrinogen and D‐dimer.^7^ However, unlike disseminated intravascular coagulation resulting from bacterial sepsis,^8^ there is minimal impact on the APTT/PT time and very mild thrombocytopenia.^7^, ^9^ Nonetheless, these individuals are predisposed to excessive fibrin deposition, particularly within the pulmonary vasculature. Autopsy findings reveal evidence of thrombi in pulmonary arterioles and arteries with diffuse alveolar damage and hyaline membrane deposition,^10^ even in patients with no clinical features of thromboembolism.^11^ Dysregulation of hemostasis in patients with COVID‐19 is closely connected to the inflammatory state, consequential to the cytokine storm and endotheliopathy induced by SARS‐CoV‐2 infection.^12^, ^13^ The natural anticoagulant properties of the endothelium, such as the expression of thrombomodulin (TM) and glycocalyx, are reduced and release of nitric oxide is impaired, increasing the hypercoagulability of the blood and promoting platelet and leucocyte adhesion.^14^, ^15^

Platelets and neutrophils recruited to sites of endothelial damage become activated by multiple pathways. Neutrophils express ACE‐2 and can be directly infected by SARS‐CoV‐2, resulting in chemokine release, complement system activation and platelet assisted neutrophil activation with formation of neutrophil extracellular traps (NETS). Prominent cytokines in the inflammatory ‘storm’ include interleukin‐6 (IL‐6), tumor necrosis factor α (TNF‐α) and C‐reactive protein (CRP). IL‐6 stimulates platelet production and activity, augments tissue factor expression on endothelial cells and monocytes, and promotes endothelial dysfunction.^16^, ^17^ Together, activated platelets, NETS, coagulation system dysregulation and endothelium disruption contribute to the prothrombotic state and resultant fibrin deposition.

D‐dimer levels reflect both thrombin generation and proteolytic degradation of fibrin by plasmin. The high D‐dimer levels in patients with COVID‐19 indicate activation of the fibrinolytic system, but despite this thrombosis predominates, indicating that a suboptimal fibrinolytic response may underlie these prothrombotic changes in COVID‐19.^18^ Generation of plasmin is governed by the balance of the plasminogen activators, tissue plasminogen activator (tPA) and urokinase (uPA), and the main inhibitors of the system, plasminogen activator inhibitor‐1 (PAI‐1), α_2_ antiplasmin (α_2_AP) and the activated form of thrombin activatable fibrinolysis inhibitor (TAFI). Circulating PAI‐1 exists in complex with the plasma glycoprotein, vitronectin, which acts to stabilize the inhibitor^19^, ^20^ and localize it within fibrin.^21^ Levels of PAI‐1 are low in the circulation, but it is released from the endothelium, circulating platelets and adipocytes following stimulation.^22^ Studies have indicated that the balance of fibrinolytic activators and inhibitors are altered in COVID‐19, however there is inadequate consensus on the drivers of the hypofibrinolytic state, with the suggestion that elevated fibrinogen,^23^, ^24^ TAFI,^24^, ^25^ tPA^26^, ^27^ and PAI‐1^24^, ^28^, ^29^ or decreased plasminogen^28^, ^30^ are responsible.

Herein, we investigate the fibrinolytic response in patients hospitalized with COVID‐19 disease compared with non‐COVID‐19 respiratory infections and a healthy cohort. We determine that COVID‐19 infection is associated with impaired plasmin generation and fibrinolysis, primarily due to elevated levels of PAI‐1 which is associated with disease severity. Importantly, we show that strategies to overcome functional PAI‐1 in COVID‐19 disease may have therapeutic potential to ameliorate persistence of fibrin and thrombosis in severely ill patients.

## 2 METHODS

### 2.1 Study design

A prospective observational cohort study was performed at Aberdeen Royal Infirmary and approved by Regional Ethics Committee (reference 16/NS/0055). Adult patients (≥18 years) presenting between April 2020 and March 2021 with acute symptoms and signs of COVID‐19, or who developed COVID‐19 while in hospital, were included after obtaining informed consent to collect blood samples and access clinical data. Samples were taken as close to presentation as the consent process permitted and patients categorized as SARS‐CoV‐2 positive or negative on the basis of PCR testing. Patients were followed up until death or discharge and samples correlated to subsequent disease progression. Ethical approval to collect blood from 30 healthy volunteers was obtained from the University of Aberdeen College Ethical Review Board, CERB/2017/9/1411.

### 2.2 Clinical data collection

For all patients, baseline premorbid demographics were extracted systematically, including, age, sex, body mass index (BMI), ethnicity, smoking status, known risk factors and medications. Dates of symptom onset and SARS‐CoV‐2 PCR test results were recorded along with presentation blood parameters taken for standard care. Data sources included patient paper and electronic hospital records, electronic hospital and primary care clinical summaries, TrakCare and patient reports. Consenting patients and/or their clinical records were reviewed daily during admission and an ordinal score representing the highest level of respiratory (and other organ) support recorded (Table 1). The maximum level of clinical support during hospital admission was grouped into mild—no oxygen support; moderate—oxygen delivered by face mask or normal nasal cannula; or severe—oxygen delivered through high flow nasal cannula (HFNC) or continuous positive airways pressure (CPAP) systems, invasive ventilation and above. Clinical notes and imaging results were reviewed for evidence of thromboembolism (venous or arterial) during admission, defined as high clinical suspicion (with or without bedside ultrasound evidence) or radiologically proven (consultant radiologist report).

**TABLE 1:** Ordinal score of level of respiratory support

Ordinal scale	Level of respiratory support
1	No oxygen support
2	Oxygen support
3	HFNC
4	CPAP
5	Ventilated
6	Ventilated with MOF

Abbreviations: CPAP, continuous positive airways pressure; HFNC, high flow nasal cannula oxygen therapy; MOF, multiorgan failure.

### 2.3 Laboratory sample preparation and handling

Blood samples were collected in 0.1 ml volume of 0.13 M trisodium citrate. Platelet poor plasma was obtained by centrifugation of whole blood samples at 2500 × *g* for 30 min at 4°C. Pooled normal plasma (PNP) was prepared as described^31^ or kindly provided by National Institute for Biological Standards and Control (NIBSC).

### 2.4 Determination of plasma protein concentrations

Concentrations of D‐dimer, PAI‐1, CRP, transforming growth factor β1 (TGF‐β1), IL‐1β, IL‐6, IL‐8, TNF‐α, uPA and leptin in plasma were determined using Simple Plex assays on Ella system, according to manufacturers' guidelines. Fibrinogen, tPA, plasminogen, TAFI, vitronectin concentrations and PAI‐1 activity were quantified using ELISA from Molecular Innovations and soluble thrombomodulin (sTM) from R&D systems.

### 2.5 Turbidity assays

Clots were formed in Tris buffered saline with Tween‐20 (TBST; 10 mM Tris, 140 mM NaCl and 0.01% Tween‐20 pH 7.4) from 30% PNP, patient or individual healthy volunteer plasma as described^32^ ± 300 pM tPA (alteplase, Actilyse) or (tenecteplase, Metalyse). Absorbance readings at 405 nm were taken every min for 8 h at 37°C on a Multiskan microplate photometer (Thermo Scientific).

### 2.6 Plasmin generation assays

Plasmin generation in plasma (10%) was measured using 0.5 mM S‐2251 (Chromogenix) ± 10 nM tPA (alteplase) and Cyanogen bromide fibrinogen fragments (10 μg/ml; Technoclone). Absorbance readings at 405 nm were taken every 30 s for 8 h at 37°C on a Biotek Flx800 microplate reader.

### 2.7 Confocal microscopy of fibrin structure

Plasma (30%) with 0.25 μM AlexaFluor 488 (AF488) or 546 (AF546) fibrinogen in TBST, CaCl_2_ (10 mM) and thrombin (0.125 U/ml) was added to Ibidi VI 0.4 slides and the clots formed for 2 h at ambient temperature. Images were recorded on Zeiss 710 laser scanning confocal microscope with a 63 × 1.40 oil immersion objective using Zeiss Zen 2012 software.

### 2.8 Western blotting

COVID‐19 positive plasma samples and PNP were separated on 4–12% NuPAGE Bis‐Tris gels under non‐reducing conditions, then transferred to PVDF membrane. PAI‐1 was detected with polyclonal PAI‐1 antibody (Affinity Biologicals) and donkey anti‐sheep IgG conjugated to HRP (Sigma).

### 2.9 Statistical analysis

Statistical analysis was performed using GraphPad Prism Software (Version 9.1.0). Normally distributed data are expressed as mean ± standard deviation and statistical significance tested using an unpaired Student's *t*‐test. Otherwise, data are expressed as median ± interquartile range (IQR) and either a Mann Whitney test or a Kruskal‐Wallis test followed by a Dunn's multiple comparison test. Statistical significance for categorical data were analyzed using a Fisher's exact test. A value of *p* < .05 was considered statistically significant for all tests. Correlations were determined by Pearson correlation coefficients. Rate of plasmin generation was calculated using; Longstaff C, 2016, Shiny App for calculating zymogen activation rates, version 0.6 (https://drclongstaff.shinyapps.io/zymogenactnCL/). Confocal microscopy images were analyzed using ImageJ 1.51w and the Diameter J^33^ plugin.

## 3 RESULTS

Of the 137 patients presenting with suspected COVID‐19 disease, 113 were positive by PCR. The remaining 24 tested negative and were managed for non‐COVID‐19 respiratory infection (Table S1). The baseline characteristics of the two groups were largely similar, although BMI was higher in the COVID‐19 cohort (Table 2).

**TABLE 2:** Patient demographics on presentation

	COVID‐19 positive (113)	Non‐COVID‐19 respiratory infection (24)	*p*
Age, mean (±SD)	60.8 (±12.6)	64.4 (±18.3)	ns
Sex (%)			
Male	66 (58.4)	10 (41.7)	ns
Female	47 (41.6)	14 (58.3)	
BMI, mean (±SD)	31.9^a^ (±6.3)	28.5 (6.9)	.02
Underweight < 18.5 (%)	0	1 (4.2)	
Normal 18.5–24.9 (%)	11 (9.7)	7 (29.2)	
Pre‐obesity 25.0–29.9 (%)	32 (28.3)	5 (20.8)	
Obesity class I 30.0–34.9 (%)	35 (30.9)	5 (20.8)	
Obesity class II 35.0–39.9 (%)	24 (21.2)	5 (20.8)	
Obesity class III > 40 (%)	10 (8.8)	1 (4.2)	
Smoker (%)			
Yes	6 (5.3)	7 (29.2)	.002
At presentation			
Anticoagulant (%)	15 (13.3)	5 (20.8)	ns
Apixaban	2	3	
Fondaparinux	1	0	
Rivaroxaban	6	1	
Dabigatran	1	0	
Edoxaban	0	1	
Warfarin	5	0	
Antiplatelet (%)	24 (21.2)	6^a^ (25)	ns
Aspirin	14	3	
Clopidogrel	8	2	
Dual—asprin and clopidogrel	2	0	
Dual—aspirin and ticagrelor	2	0	
ACE inhibitors/ARB (%)	36 (31.9)	4 (16.7)	ns
Medical history (%)			
DVT/PE	8 (7.1)	2 (8.3)	ns
CVA/TIA	8 (7.1)	1 (4.2)	ns
IHD	22 (19.5)	5 (20.8)	ns
PVD	5 (4.4)	1 (4.2)	ns
Heart failure	8 (7.1)	2 (8.3)	ns
Hypertension	36 (31.9)	7 (29.2)	ns
AF	0	0	ns
MI	0	0	ns
COPD/asthma	29 (25.6)	10 (41.7)	ns
Pulmonary other	9 (8.0)	2 (8.3)	ns
DM	28 (24.8)	5 (20.8)	ns
Immunosuppression	15 (13.3)	4 (16.7)	ns
CKD ≥ 3	10 (8.8)	4 (16.7)	ns
Liver disease	4 (3.5)	0	ns
Days hospitalized median (IQR)	7 (4.0–11)	3 (1.0–7.8)	˂.001
Respiratory support (%)			
No oxygen support	16 (14.2)	13 (54.2)	˂.001
Oxygen support	70 (61.9)	10 (41.7)	ns
HFNC/CPAP	22 (19.45)	0	ns
Ventilated	5 (4.4)	0	ns
Thrombotic events	4	0	ns
Deaths (%)	18 (15.9)	2 (8.3)	ns
COVID‐19 as cause of death (%)	17 (15.0)	n/a	

*Note:* Quantitative data were analyzed by an unpaired Student's *t*‐test and expressed as mean ± SD or analyzed with a Mann–Whitney test and expressed as median (IQR). Statistical significance for categorical data was analyzed using a Fisher's exact test.

Abbreviations: ACE, angiotensin‐converting enzyme; AF, atrial fibrillation; ARBs, inhibitors and angiotensin II receptor blockers; BMI, body mass index; CKD, chronic kidney disease; COPD, chronic obstructive pulmonary disease; DM, diabetes mellitus; DVT, deep vein thrombosis; IHD, ischemic heart disease; MI, myocardial infarction; PE, pulmonary embolism; PVD, peripheral vascular disease.

^a^ Indicates missing data for some patients.

The median hospital stay of the COVID‐19 cohort was significantly longer than those with non‐COVID‐19 respiratory infection (7 [IQR 4–11] vs. 3 [IQR 1–7.8] days, Table 2) but the death rate, although higher (15.9% vs. 8.3%), did not reach significance. The mean age of the COVID‐19 patients was 60.8 ± 12.6 years, 66 (58.4%) were male and four (3.5%) experienced a thrombotic event (three PE and one ischemic stroke), of whom one required mechanical ventilation (Table 2). Presentation full blood counts (Table 3) showed significantly reduced leucocytes compared with patients with non‐COVID‐19 respiratory infections and reduced platelet counts, although these did not reach significance.

**TABLE 3:** Presentation full blood counts

	COVID‐19 positive (113)	Non‐COVID‐19 respiratory infection (24)	*p*
Hemoglobin (g/L)	140.0 (129.0–151.0)	130.5 (112.3–151.0)	ns
Platelets (×10^9^/L)	213.5 (174.0–269.3)	243.0 (206.3–298.3)	ns
Leucocyte (×10^9^/L)	6.6 (4.9–8.4)	12.2 (9.3–16.0)	<.001
Neutrophils (×10^9^/L)	5.1 (3.5–6.5)	10.2 (7.0–13.8)	<.001
Lymphocytes (×10^9^/L)	0.8 (0.6–1.3)	1.2 (7.0–13.8)	ns
Eosinophils (×10^9^/L)	0.0 (0–0.02)	0.06 (0.0–0.18)	<.001
Basophils (×10^9^/L)	0.0 (0–0.01)	0.02 (0.01–0.05)	.003
Monocytes (×10^9^/L)	0.5 (0.3–0.6)	0.8 (0.4–1.2)	<.001

*Note:* Data are presented as median (IQR). Statistical difference was determined by Mann–Whitney test.

### 3.1 Fibrinolytic antigens are increased in patients with COVID‐19 and associate with clinical inflammatory biomarkers

**FIGURE 1:**
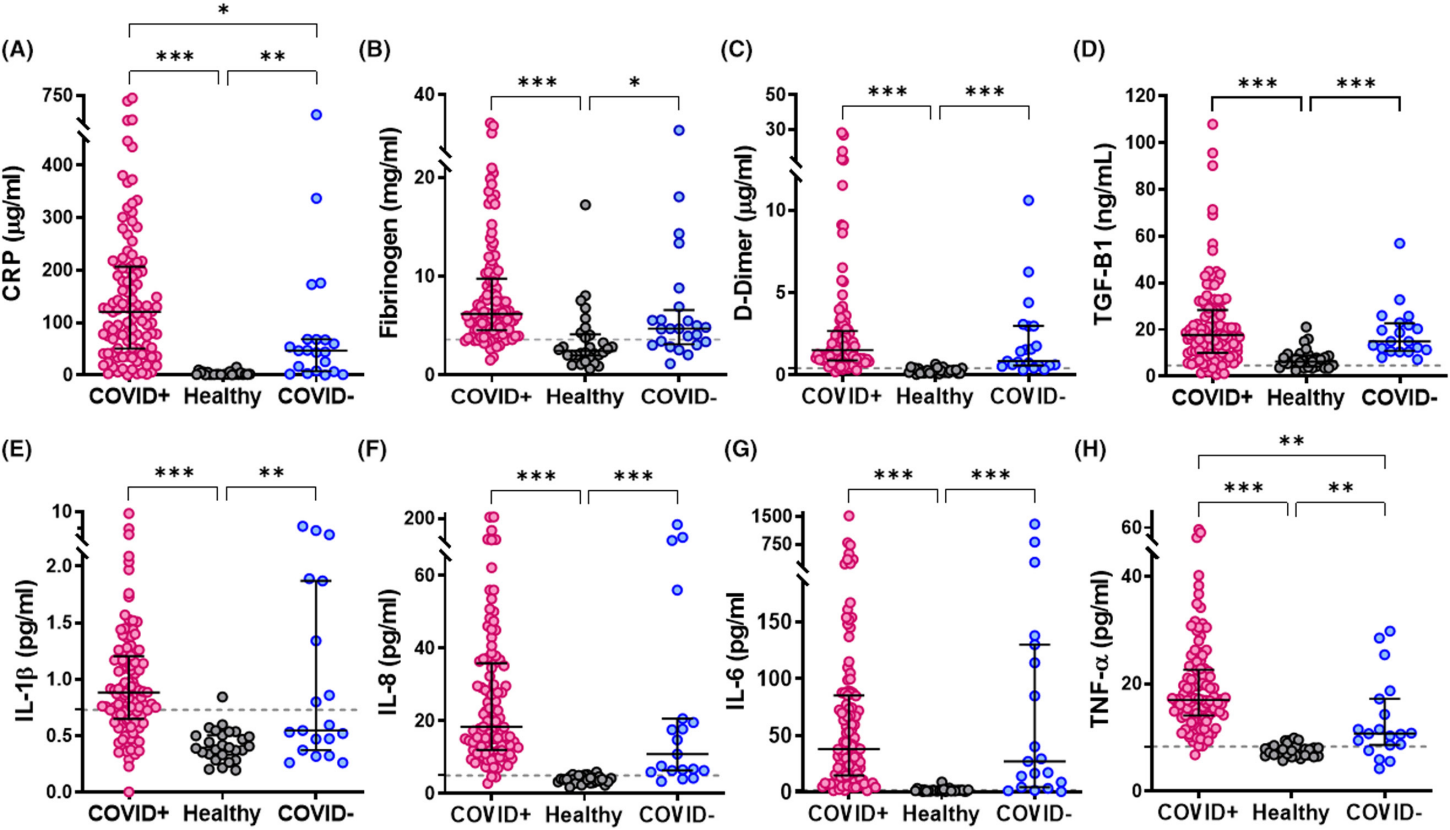
Inflammatory markers and proinflammatory cytokines in COVID‐19. Antigen levels of inflammatory markers and proinflammatory cytokines were measured in plasma from confirmed COVID‐19 patient plasma (COVID+), other non‐COVID‐19 respiratory infections (COVID−) or healthy controls by Simple Plex assays on Ella system or by ELISA for fibrinogen. (A) C‐reactive protein (CRP), (B) fibrinogen, (C) D‐dimer, (D) transforming growth factor β1 (TGF‐β1), (E) interleukin (IL)‐1β, (F) IL‐6, (G) IL‐8 and (H) tumor necrosis factor α (TNF‐α). Dotted lines indicate antigen concentrations in pooled normal plasma. Data are shown as median ± interquartile range (IQR), *p < .05, **p < .01 and ***p < .001.

We initially quantified several clinical diagnostic markers of inflammation and coagulation. CRP was significantly elevated in patients with COVID‐19 compared with healthy controls (*p* < .001) and non‐COVID‐19 respiratory infection (*p* < .05, Figure 1A). Fibrinogen (*p* < .001, Figure 1B) and D‐dimer (*p* < .001, Figure 1C) levels were raised in COVID‐19 disease but only reached significance compared with healthy controls. In line with the well documented ‘cytokine storm’ in COVID‐19, we observed significantly elevated levels of TGF‐β1, IL‐1β, IL‐8, IL‐6 and TNF‐α (Figure 1D‐H) compared with healthy controls. TNF‐α was significantly higher in patients with COVID‐19 compared with non‐COVID‐19 respiratory infection (*p* < .01, Figure 1G).

**FIGURE 2:**
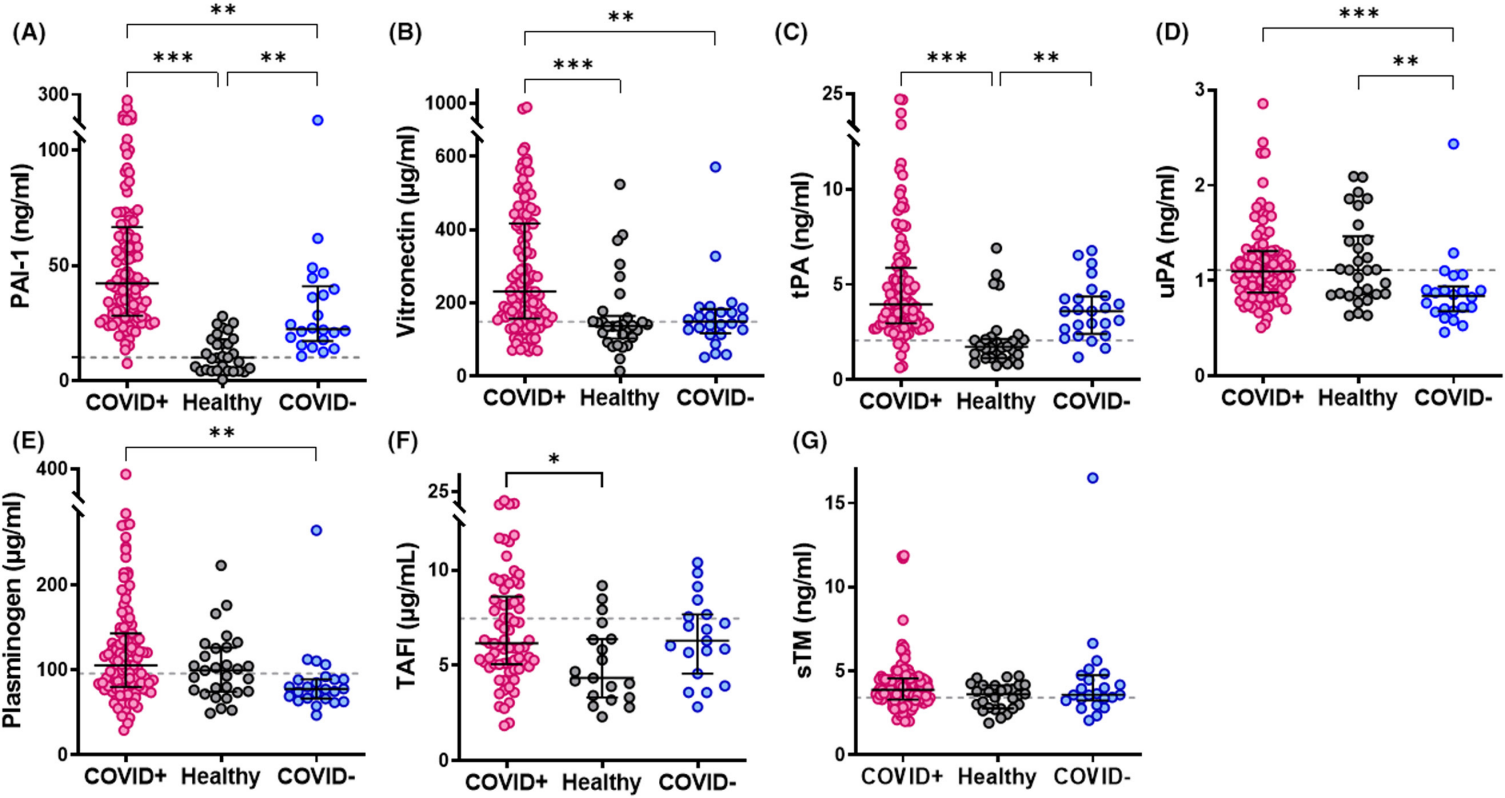
Fibrinolytic proteins are elevated in COVID‐19. The concentrations of fibrinolytic proteins were measured in plasma from confirmed COVID‐19 patient plasma (COVID+), other non‐COVID‐19 respiratory infections (COVID−) or healthy controls by Simple Plex assays on Ella system or by ELISA for plasminogen activator inhibitor‐1 (PAI‐1). (A) PAI‐1, (B) vitronectin, (C) tissue plasminogen activator (tPA), (D) urokinase (uPA) (E) plasminogen, (F) thrombin activatable fibrinolysis inhibitor (TAFI) and (G) soluble thrombomodulin (sTM). Dotted lines indicate antigen concentrations in pooled normal plasma. Data are shown as median ± interquartile range (IQR), *p < .05, **p < .01 and ***p < .001.

PAI‐1 and its cofactor vitronectin were significantly elevated in patients with COVID‐19 compared with healthy controls (*p* < .001) and non‐COVID‐19 respiratory infection (*p* < .01) (Figure 2A,B). We observed bands in COVID‐19 plasma at a molecular mass consistent with the size of the reported PAI‐1‐Vn complex^19^ (Figure S1). Of note, tPA and TAFI levels were elevated in patients with COVID‐19 disease compared with healthy controls (*p* < .001 and *p* < .05, respectively) but were not different compared with non‐COVID‐19 respiratory infection (Figure 2C,F). Interestingly, plasminogen differed only between the two disease groups due to a reduced level in non‐COVID respiratory disease (*p* < .01), with uPA also attenuated in this disease group (Figure 2D,E). There was no significant difference in sTM (Figure 2G).

The relationship between inflammatory and fibrinolytic markers with COVID‐19 disease progression was studied by grouping according to the level of maximum oxygen support required. CRP and D‐dimer increased with disease severity, with CRP being a strong indicator of progression to more severe COVID‐19 illness (Figure S2A,B). IL‐6 and IL‐8 also increased with disease progression (Figure S2D,E). PAI‐1 and tPA were the only fibrinolytic proteins that stratified with escalating severity of COVID‐19 (Figure S3A,D). Changes in PAI‐1 activity did not reach statistical significance, potentially reflecting the difference in quantifying free versus total PAI‐1.

PAI‐1 antigen was found to correlate strongly with TGF‐β1 (*r* = .39, *p* < .001), IL‐8 (*r* = .58, *p* < .001), moderately with IL‐1β (*r* = .31, *p* < .01) and TNF‐α (*r* = .33, *p* < .001). Additionally, a weak correlation was observed between PAI‐1, fibrinogen (*r* = .27, *p* < .01) and D‐dimer (*r* = .23, *p* < .05) but not with CRP (Figure S4). PAI‐1 levels did not correlate with BMI in COVID‐19 disease (*r* = .039, *p* = .69), despite the documented relationship with obesity. Leptin levels, which are known to correlate with body fat, were not different between the patient groups and there was no relationship with PAI‐1 (Figure S5). Nonetheless, leptin significantly correlated with BMI in patients with COVID‐19 (Figure S5).

### 3.2 Suboptimal fibrinolytic potential and altered fibrin clot structure in COVID‐19 disease

**FIGURE 3:**
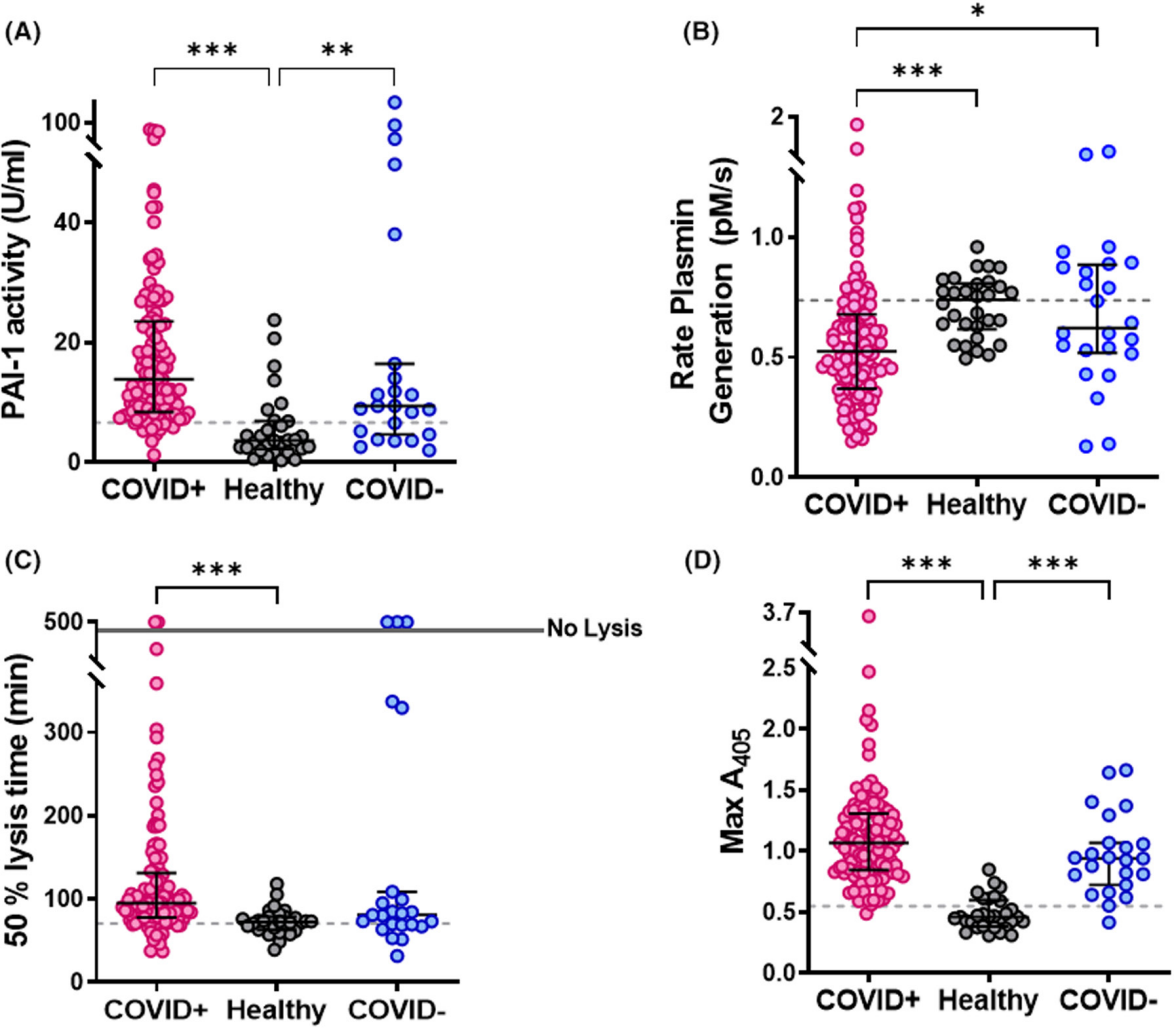
COVID‐19 disease is associated with reduced fibrinolytic potential. (A) Active plasminogen activator inhibitor‐1 (PAI‐1) was measured using a PAI‐1 chromogenic activity assay. Data are presented as the median activity (U/ml) ± IQR. (B) Plasmin generation in plasma (10%) was measured using S‐2251 (0.5 mM) in the presence or absence of 10 nM alteplase with CBNR fibrinogen fragments (10 μg/ml) included to stimulate tissue plasminogen activator (tPA) activity. The absorbance at 405 nm was read every 30 s for 8 h at 37°C. Data are the average rate of plasmin generation (pM/s) ± IQR. (C) Plasma clots (30%) were formed with plasma from patients or healthy volunteers in the presence phospholipids (16 mM) ± alteplase (300 pM). Clotting was initiated with CaCl_2_ (10.6 mM) and thrombin (0.1 U/ml) and absorbance readings were taken every min for 8 h at 37°C. Data show the median ± IQR time to 50% lysis and (D) maximum absorbance of plasma clots at 405 nm. *p < .05, **p < .01 and ***p < .001.

We then addressed the functional impact of changes in the balance of activators and inhibitors in COVID‐19 disease. Elevated PAI‐1 antigen correlated with an increase in PAI‐1 activity (*r* = .55, *p* < .001), which was 3.8‐fold higher in the plasma from patients with COVID‐19 compared with healthy controls (Figure 3A, *p* < .001). The rate of tPA‐mediated plasmin generation (Figure 3B, *p* < .001) was significantly attenuated compared with patients with non‐COVID‐19 disease and healthy controls, giving rise to slower rates of clot lysis (Figure 3C; *p* < .001). A positive correlation existed between clot lysis time and PAI‐1 antigen (*r =* .3, *p* = .001) and activity (*r* = .74, *p* < .001) in patients with COVID‐19 (Figure S6). Levels of fibrinogen and D‐dimer also correlated with PAI‐1 activity levels (*r* = .27, *p* = <.001; *r* = .23, *p* < .05, respectively; Figure S6).

**FIGURE 4:**
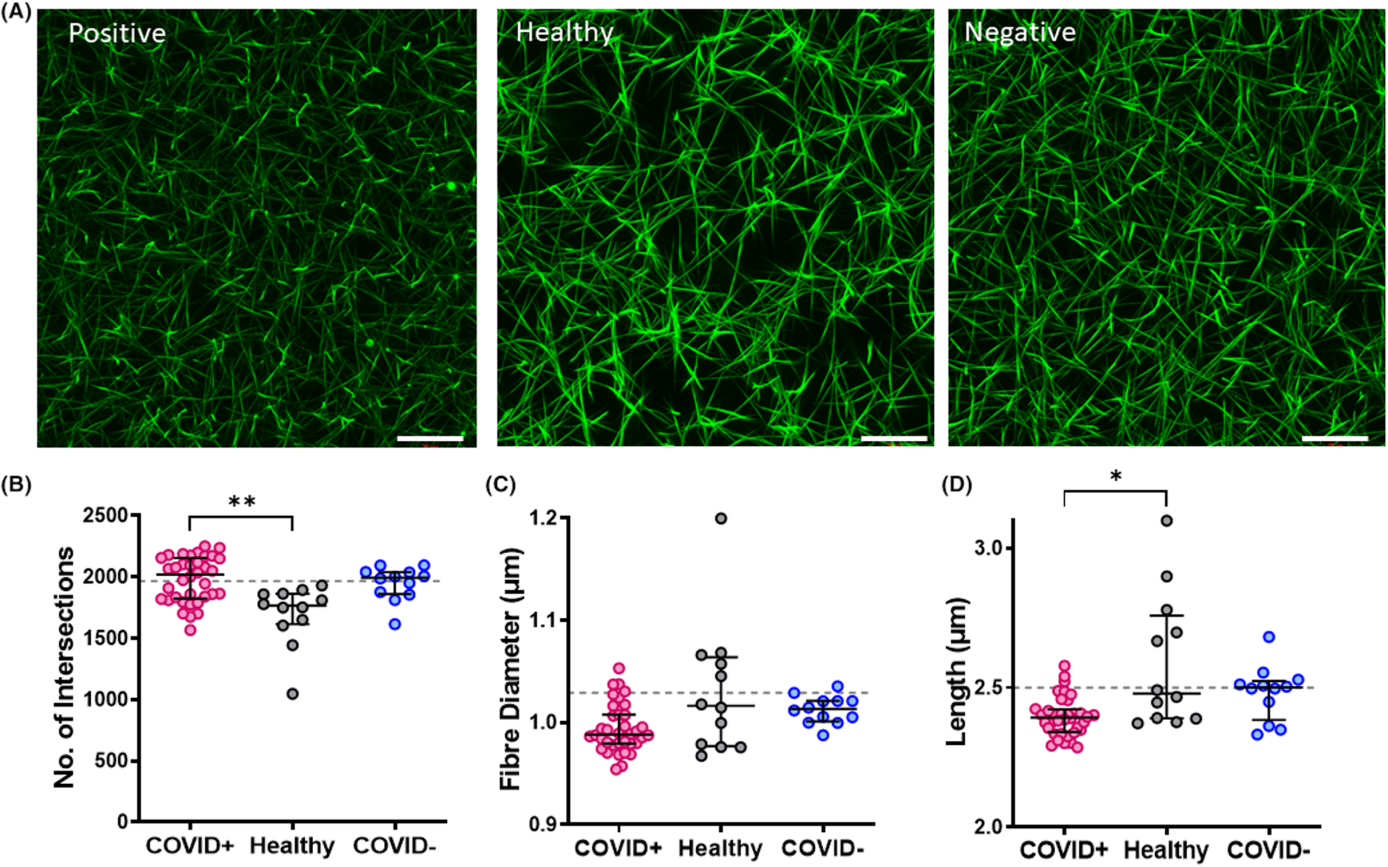
Clots formed from COVID‐19 plasma exhibit a denser fibrin network. Plasma clots (30%) were formed in the presence of AF488 or AF546 labeled fibrinogen (0.25 μM) by initiating clotting with CaCl_2_ (10 mM) and thrombin (0.125 U/ml) in Ibidi VI 0.4 μ slides. After allowing to clot for 1 h images were recorded on Zeiss 710 laser scanning confocal microscope with a 63 × 1.40 oil immersion objective using Zeiss Zen 2012 software. (A) Representative images of clots formed from plasma of a patient who was COVID‐19 positive (COVID+), healthy donor and non‐COVID‐19 respiratory infections (COVID−). (B) Quantification of the number of intersections, which is indicative of branching and clot density. (C) Quantification of fiber diameter (D) Quantification of mean fibrin fiber length. Representative images of *n* ≥ 3. Scale bar indicates 20 μm. *p < .05 and **p < .01.

The median maximum absorbance of plasma clots from the COVID‐19 and non‐COVID‐19 respiratory infection was significantly higher than healthy controls (Figure 3D; *p* < .001). Higher maximum absorbance can signify changes in fibrin clot structure and/or be reflective of baseline fibrinogen levels. Maximum absorbance correlated with fibrinogen (*r* = .29, *p* < .01; Figure S6) and vitronectin (*r* = .33, *p* = .001; Figure S7) in COVID‐19 disease. Analysis of fibrin clot structure by confocal microscopy revealed a denser fibrin network with more branching, indicated by the increased number of intersections (Figure 4A,B, *p* < .05) in patients who were COVID‐19 positive compared with healthy controls. Fibrin fibers were also thinner (Figure 4A,C) and significantly shorter (Figure 4A,D, *p* < .01).

### 3.3 Elevated PAI‐1 drives the hypofibrinolytic state in COVID‐19 disease

**FIGURE 5:**
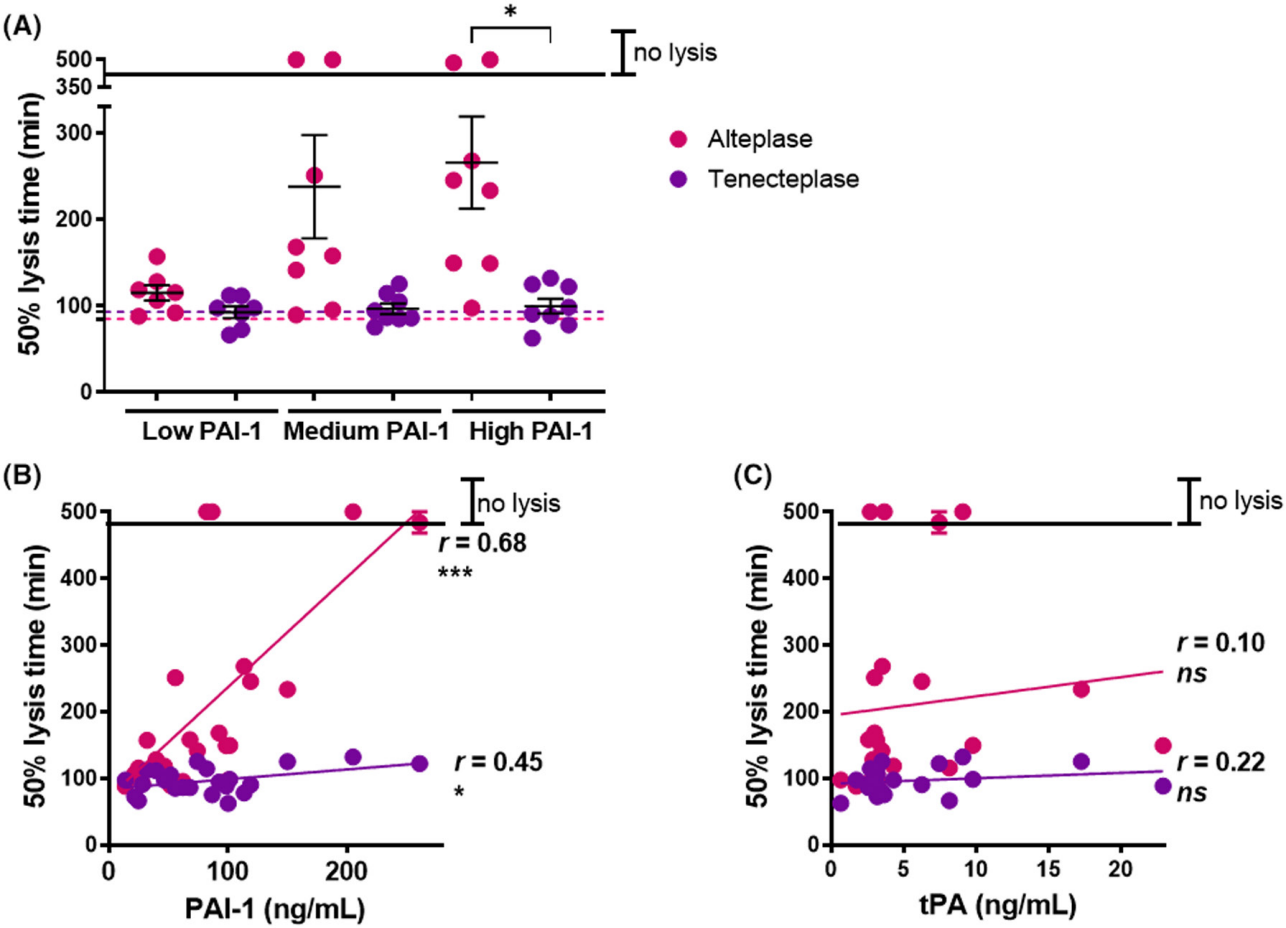
The tissue plasminogen activator (tPA) variant, tenecteplase, overcomes the reduced fibrinolytic potential in COVID‐19 plasma. (A) Plasma clots (30%) were formed with COVID‐19 plasma with low <50 ng/ml (13.4–46.4 ng/ml); medium 50–100 ng/ml (51.5–92.3 ng/ml) or high >100 ng/ml (103.3–260 ng/ml) PAI‐1 antigen in the presence of phospholipids (16 mM) ± alteplase or tenecteplase (300 pM). Clotting was initiated with CaCl_2_ (10.6 mM) and thrombin (0.1 U/ml) and absorbance readings were taken every min for 8 h at 37°C. Data show the median ± IQR time to 50% lysis of plasma clots at 405 nm. (B) Correlation of 50% lysis time with PAI‐1 antigen (C) or tPA antigen. *p < .05 and ***p < .001.

To determine the direct contribution of PAI‐1 in the reduced fibrinolytic potential in COVID‐19 disease we utilized tenecteplase, a variant of tPA that is resistant to inhibition by PAI‐1. Plasma clots were initially formed with PNP with various amounts of purified PAI‐1 and lysed with alteplase versus tenecteplase. A dose‐dependent increase in clot lysis time was observed with alteplase on addition of PAI‐1, however lysis by tenecteplase was unaffected by addition of the inhibitor (Figure S8A). A subset of the COVID‐19 samples were stratified according to their PAI‐1 antigen levels (low <50 ng/ml [13.4–46.4 ng/ml]; medium 50–100 ng/ml [51.5–92.3 ng/ml]; high >100 ng/ml [103.3–260 ng/ml]) and clots lysis studies performed with alteplase and tenecteplase. At low PAI‐1 concentrations, clots from patients with COVID‐19 lysed at similar rates with alteplase and tenecteplase (Figure 5A & Figure S8B). In contrast, plasma clots with high PAI‐1 lysed significantly slower with alteplase compared with tenecteplase (Figure 5A & Figure S8B). A strong correlation between 50% lysis time and PAI‐1 concentration existed with alteplase (*r* = .68, *p* < .001), which was reduced with tenecteplase (*r* = .45, *p* < .05, Figure 5B). No relationship was observed between 50% lysis time and endogenous tPA antigen (Figure 5C). These data suggest that there is a shift in the balance of fibrinolysis in COVID‐19 disease towards inhibition, arising from high PAI‐1.

## 4 DISCUSSION

We examined the hemostatic dysregulation, specifically the contribution of the fibrinolytic pathway, in a large prospective cohort of patients hospitalized with COVID‐19 disease. Our findings reveal marked elevation of PAI‐1 and its stabilizing cofactor vitronectin in COVID‐19 disease compared with non‐COVID‐19 respiratory infections. High PAI‐1 suppressed plasmin generation and fibrinolysis in COVID‐19, despite concomitant increases in tPA. Stratification of COVID‐19 plasma according to PAI‐1 levels revealed delayed lysis with alteplase with increasing PAI‐1 but no difference in tenecteplase lysis. These functional data demonstrate that it is the high PAI‐1 in COVID‐19 disease that dictates the suboptimal fibrinolytic response. PAI‐1 levels were associated with several inflammatory cytokines known to be elevated in COVID‐19 disease, including IL‐8, Il‐6, TGF‐β1 and TNF‐α. Interestingly, higher levels of PAI‐1 were associated with a poorer clinical outcome, suggesting that it may be a useful therapeutic target and/or a potential biomarker to help stratify those at risk of developing more severe COVID‐19.

Plasminogen activators circulate at low concentrations, while PAI‐1 and α_2_AP exist in molar excess, preventing unrestricted plasmin activity. The primary source of tPA in the circulation is vascular endothelial cells, with release in response to specific stimuli, including bradykinin and TNF‐α. Other hemostatic proteins derived from the endothelium, including P‐selectin, vWF and factor VIII, are upregulated in COVID‐19 disease.^34^ We found significantly elevated levels of tPA in patients with COVID‐19 compared to healthy controls, but not to other non‐COVID‐19 respiratory infections. However, the levels reported herein are several fold lower than those described by Zuo and colleagues,^26^ perhaps reflecting differences in methodology and/or patient cohort. We observed an association between increasing levels of tPA and COVID‐19 disease severity, similar to observations in a smaller cohort.^35^ Interestingly, plasminogen and uPA levels were not altered in COVID‐19 disease but were reduced in non‐COVID‐19 respiratory infections. A longitudinal study of 14 patients with COVID‐19 indicated a reduction in plasminogen with increasing COVID‐19 disease severity,^28^ but we did not observe significant differences in plasminogen in this large cohort of patients stratified according to severity.

Fibrinogen and thrombin concentration directly impact the quality of the fibrin network, with dense networks composed of thin fibers being directly associated with the pathophysiology of arterial and venous thromboembolic complications.^36^ We describe elevated levels of fibrinogen in COVID‐19 disease, as previously reported.^23^, ^24^, ^37^ Similar to a recent publication, we found evidence of a dense fibrin network composed of thinner and shorter fibers with increased numbers of branching points.^37^ Given the clear relationship between compact clot structure and down‐regulation of fibrinolysis,^36^ abnormal structure may contribute to persistence of fibrin within the microenvironment of the lung and systemically in critically ill individuals with COVID‐19 disease.

Our work demonstrates a marked decrease in fibrinolytic potential in COVID‐19 disease, observed as a reduction in plasmin generation and fibrinolysis, despite elevated tPA. These results contrast a previous report comparing COVID‐19 disease and sepsis, which showed increased endogenous plasmin potential in patients with COVID‐19.^38^ However, this group also reported an increase in PAI‐1 activity in COVID‐19, in line with our observations.^39^ Elevated levels of the fibrinolytic inhibitors, PAI‐1 and TAFI in COVID‐19 disease may promote the suboptimal fibrinolytic response in these individuals. TAFI was elevated in our cohort of patients with COVID‐19 compared with healthy individuals, yet there was not a concomitant increase in its cofactor, TM. These observations were surprising, given other reports on increased levels of sTM due to endothelialopathy in COVID‐19 disease.^40^ In contrast, we show marked elevation of PAI‐1 and its cofactor vitronectin in COVID‐19 disease. Vitronectin serves not only to extend the half‐life of circulating PAI‐1^19^, ^41^ but importantly localizes this serpin within the fibrin network.^21^ It is this latter aspect that is suspected to confer the enhanced fibrinolytic resistance as PAI‐1 inhibition of fibrin clot lysis is dependent on the concentration of vitronectin.

To delineate the role of PAI‐1 in driving the suboptimal fibrinolytic state in COVID‐19 disease we compared lysis of plasma clots from patients with COVID‐19 stratified according to PAI‐1 levels. A direct correlation was observed between alteplase lysis time and PAI‐1 concentration in COVID‐19 plasma. Use of tenecteplase, a variant of alteplase with an 80‐fold reduction in sensitivity to PAI‐1,^42^ was able to restore the fibrinolytic potential in plasma from patients with COVID‐19 with high PAI‐1 levels. These data directly attribute the suboptimal fibrinolytic response in patients with COVID‐19 to PAI‐1 that is likely sustained by elevated vitronectin. Therapeutic use of alteplase has gained interest as an intervention to target fibrin deposition in patients with COVID‐19.^43^, ^44^ Our data show the superiority of tenecteplase to overcome PAI‐1 in COVID‐19 disease and suggest that the clinical use of this variant as a thrombolytic may have improved benefit over alteplase.

Plasma PAI‐1 can be derived from platelets, the vascular endothelium and adipocytes. The source of plasma PAI‐1 in COVID‐19 disease is unclear, but it has been suggested to relate to increased obesity in severely ill patients.^18^ BMI was significantly higher in our cohort of patients with COVID‐19 compared with those with non‐COVID‐19 respiratory illness but nonetheless there was no relationship between PAI‐1 and BMI or with leptin, a biomarker of metabolic syndrome.^45^ In contrast, leptin significantly correlated with increasing BMI in the COVID‐19 cohort. These data suggest adipocytes may not be the source of PAI‐1. Platelets contain the major pool of circulating PAI‐1, which is released upon activation into the surrounding milieu and retained on the surface of activated platelets.^46^, ^47^ Our previous data have shown that vitronectin is released with PAI‐1 from activated platelets.^47^ Markers of ongoing platelet activation,^48^ including P‐selectin,^49^ are well documented in patients with COVID‐19, therefore it is plausible that degranulation of platelets could account for these increases in the plasma pool of PAI‐1 and vitronectin. Activation of the endothelium and release of PAI‐1 in response to cytokine and thrombin stimulation may also promote increased systemic levels of PAI‐1. Interestingly, both PAI‐1 and vitronectin were previously found to be elevated in SARS‐CoV, a coronavirus which emerged almost 20 years prior in 2002.^50^ Regardless of the source of increased levels of these proteins, these data indicate that the PAI‐1‐vitronectin axis may be of importance in contributing to fibrin persistence and lung damage and thereby warrants attention as a potential therapeutic target.

Fibrinolytic and proinflammatory markers including D‐dimer, CRP and fibrinogen were significantly elevated in this cohort of hospitalized patients with COVID‐19, consistent with previous reports.^7^, ^51^, ^52^, ^53^, ^54^, ^55^ Notably, only CRP reached statistical significance in patients with COVID‐19 compared with non‐COVID‐19 respiratory infection. CRP has been shown to provoke local release of PAI‐1 from endothelial cells^56^, ^57^ thereby fostering further fibrin persistence in the alveolar space. In addition, several proinflammatory cytokines promote PAI‐1 synthesis. IL‐8, which correlated strongly with PAI‐1 in this cohort, perturbs the balance of PAI‐1 and tPA in endothelial cells^58^ and may foster the high systemic PAI‐1 levels. Models of SARS‐CoV infection have demonstrated increased TGF‐β1, which in turn induces PAI‐1,^59^ which is linked to development of ARDS.^60^ We found elevated TGF‐β1 in severe COVID‐19 disease, which strongly correlates with PAI‐1. A non‐biased systems biology approach to study infection with SARS‐CoV revealed over‐expression of PAI‐1 leading to fibrin persistence.^61^ Furthermore, IL‐6, an acute‐phase protein that stimulates synthesis of both PAI‐1 and tPA,^62^ is upregulated in this cohort. Interestingly, tocilizumab, an IL‐6 antagonist, which at has been used successfully to combat severe COVID‐19 disease,^63^ reduced PAI‐1 in this group of patients providing direct evidence of the link between the proinflammatory state and PAI‐1 levels.^64^ The endotheliopathy and prothrombotic state are known to persist in patients with COVID‐19 4–12 months post‐discharge,^18^, ^65^ with elevated PAI‐1 a component of this syndrome. It remains to be seen whether therapeutic agents, like tocilizumab, that dampen the inflammatory response, could be beneficial in reducing the prothrombotic tendency of these individuals.

### 4.1 Limitations

The unprecedented nature of the COVID‐19 pandemic led to practical difficulties in taking consented research samples, however most samples were taken within 24 h of presentation or diagnosis. Administration of thromboprophylaxis is routine in hospital inpatients and those in critical care environments may have received higher prophylactic doses, mainly of low molecular weight heparin. Thrombosis outcomes were based upon attending clinical opinion and/or from radiological report. These interventions may have had a bearing on the reduced rates of death and thrombotic complications and levels of D‐dimer observed in the presented cohort compared with earlier studies, such as Tang et al.^7^ In addition, during the time frame of this study new treatment options, such as dexamethasone and tocilizumab, were introduced; however, as patients were sampled within 24 h these are unlikely to have bearing on the results.

## 5 CONCLUSIONS

Our data demonstrate that PAI‐1 is systemically elevated in COVID‐19 disease, and that there is a concomitant increase in its cofactor vitronectin which confers fibrinolytic resistance. Importantly, our functional studies, utilizing PAI‐1 resistant forms of tPA, reveal the pivotal role of PAI‐1 in dictating the suboptimal fibrinolytic response during SARS‐CoV2 infection. The reduction in fibrinolytic potential occurs despite elevated tPA, underscoring the dominant role of PAI‐1 in this setting. Our data accentuate the prognostic value of PAI‐1 and the potential to repurpose existing drugs, such as tenecteplase, to treat the hypofibrinolytic state in COVID‐19 and other acute respiratory diseases.

## AUTHOR CONTRIBUTIONS

CSW, MS and GBM designed, executed, analyzed research and wrote/edited the manuscript. CAW executed and analyzed the research. AJM, JCK, SS, NC and CNB collected samples and reviewed the manuscript. HW and JGC collected samples, analyzed clinical and laboratory data, wrote and edited the manuscript. NJM designed, executed, analyzed, managed the research and wrote/edited the manuscript.

## CONSENT STATEMENT

All authors provide consent to publication of this data.

## FUNDING INFORMATION

This research was supported by NHS Grampian Endowment (COV19‐004 and 20/021), Medical Research Scotland (CVG‐1721‐20), The University of Aberdeen Development Trust (RG15537), Friends of Anchor (RS 2019 003) and the National Institute for Health Research (NIHR) Oxford Biomedical Research Centre (BRC). The views expressed are those of the authors and not necessarily those of the NHS, the NIHR or the Department of Health. CSW and NJM are supported by the British Heart Foundation (PG/15/82/31721; PG/20/17/35050). JGC is supported by NHS Research Scotland. SS receives funding support from the Medical Research Council (MR/T024054/1).

## CONFLICT OF INTEREST

The authors have no relevant conflict of interests to declare.

## Supporting information


Appendix S1
