# Supplementary material for: The suboptimal fibrinolytic response in COVID‐19 is dictated by high PAI‐1
Source: J Thromb Haemost. 2022 Jul 21:10.1111/jth.15806. Online ahead of print. doi: 10.1111/jth.15806 (PMC9349442; doi:10.1111/jth.15806)
Supplement: Supplementary file 1 — Appendix S1 [file JTH-9999-0-s001.pdf]

Whyte CS, Simpson *et al*, 2022

Supplemental Material

Non-COVID-19 respiratory infection (24)	
LRTI/Pneumonia - Non-COVID-19	17
Viral Respiratory illness - Non-COVID-19	3
Aspiration Pneumonia	2
Exacerbation Asthma/COPD	2

Supplementary Table 1 – Breakdown of the type of Non-COVID-19 respiratory infections. Lower respiratory tract infection (LRTI), chronic obstructive pulmonary disease (COPD).

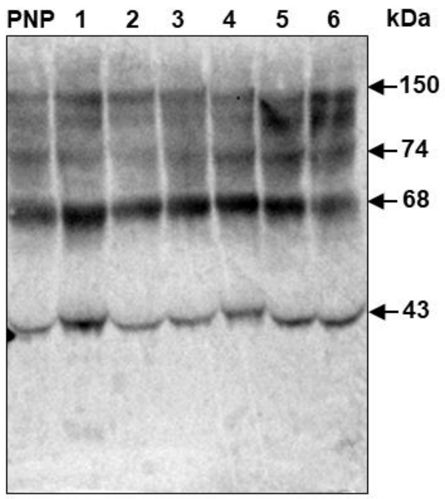

Supplementary Figure 1 – PAI-1 – Vn complex detected by Western blotting. COVID-19+ plasma samples (1-6) and pooled normal plasma (PNP) were separated on 4-12% NuPAGE Bis-Tris gels under non-reducing conditions, then transferred to PVDF membrane. PAI-1 was detected with polyclonal PAI-1 antibody (Affinity Biologicals) and donkey anti-sheep IgG conjugated to HRP (Sigma). Bands of PAI-1 protein were detected at 43, 68, 74 and 150 kDa.

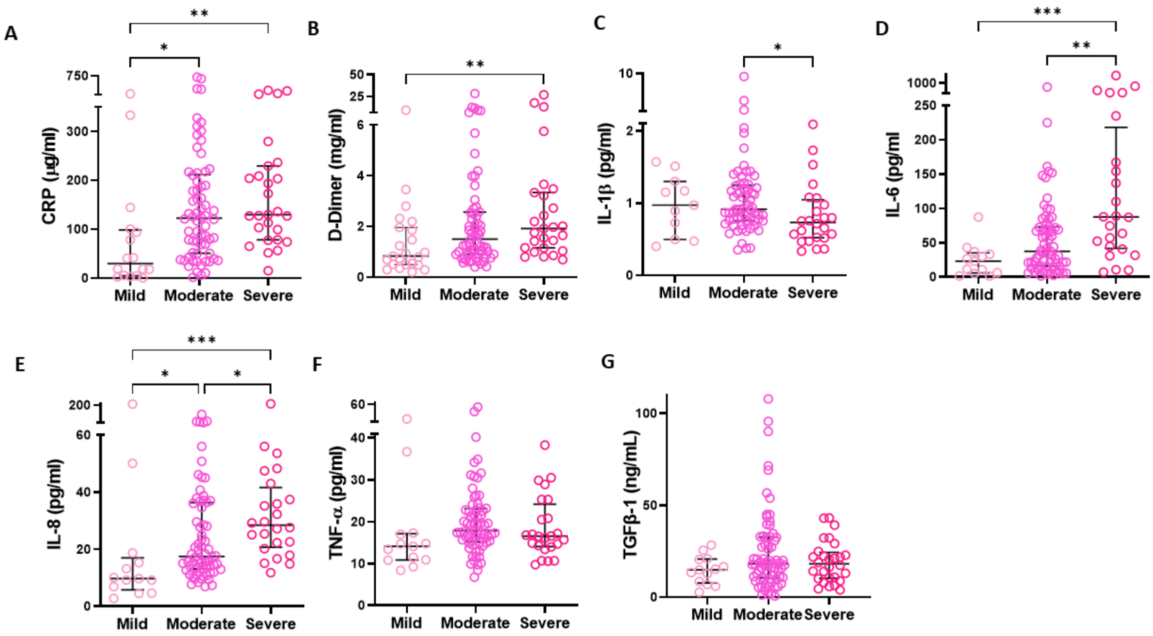

Supplementary Figure 2 – Inflammatory markers and proinflammatory cytokines increase with COVID-19 disease severity. Inflammatory markers were grouped according into the following groups of disease progression; mild = patients that did not require supplemental oxygen, moderate = patients on supplemental oxygen support or severe = patients on high flow nasal cannula (HFNC), continuous positive airway pressure (CPAP) or patients that were ventilated. (A) CRP antigen, (B) D-dimer, (C) IL-1β, (D) IL-6, (E) IL-8, (F) TNF-α and (G) TGF-β1. * $p<0.05$, ** $p<0.01$ and *** $p<0.001$.

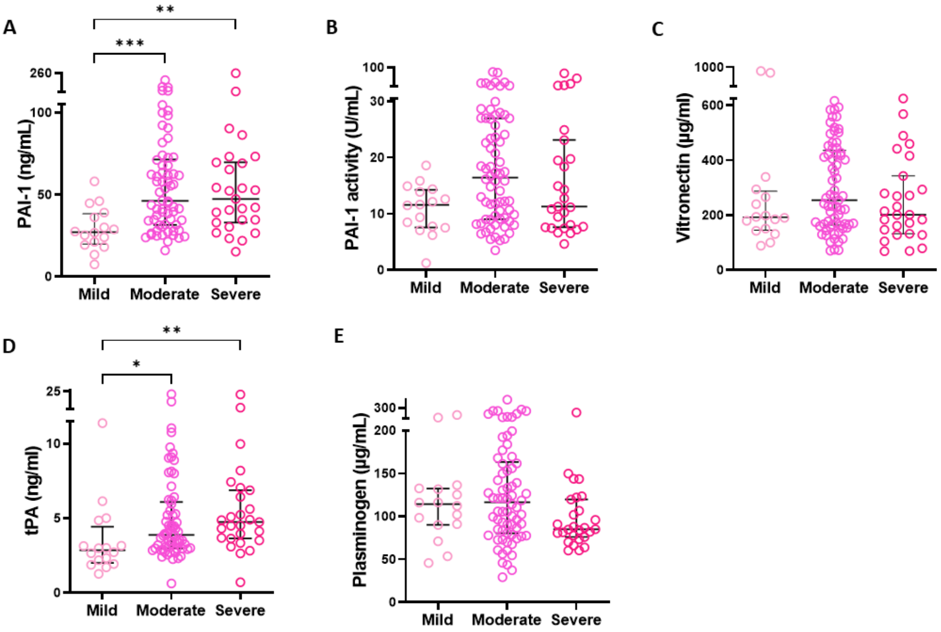

Supplementary Figure 3 – PAI-1 and tPA increase with COVID-19 disease severity. Fibrinolytic markers were grouped according to disease severity; mild = patients that did not require supplemental oxygen, moderate = patients on supplemental oxygen support or severe = patients on high flow nasal cannula (HFNC), continuous positive airway pressure (CPAP) or patients that were ventilated. (A) PAI-1 antigen, (B) PAI-1 activity, (C) vitronectin, (D) tPA antigen and (E) plasminogen. * $p<0.05$, ** $p<0.01$ and *** $p<0.001$.

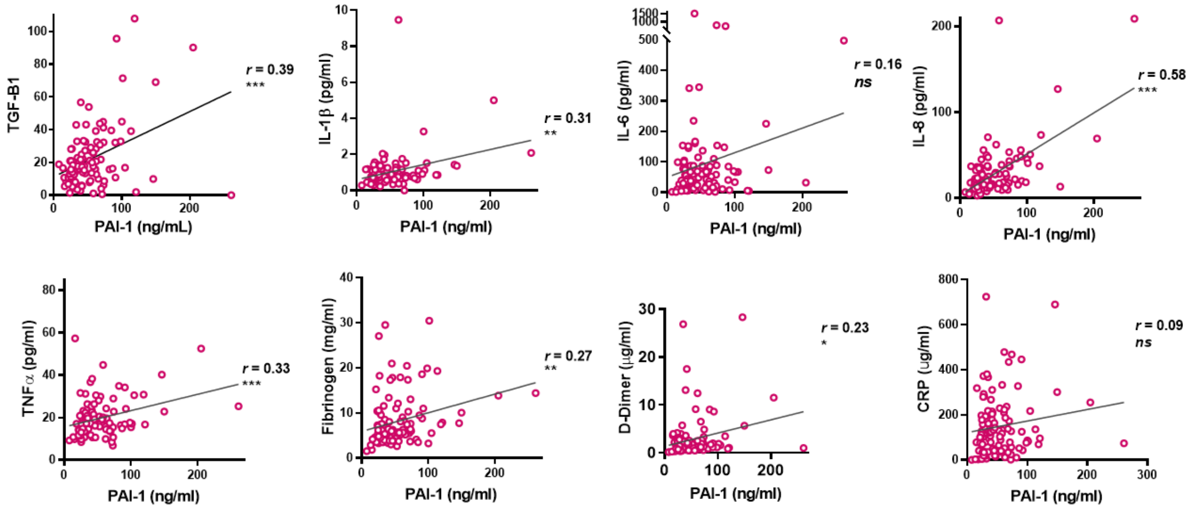

Supplementary Figure 4 – PAI-1 correlates with inflammatory markers and proinflammatory cytokines. Pearson correlation coefficients (r) were calculated between PAI-1 antigen and TGF-β1 - IL-1β, IL-6, IL-8 TNF-α fibrinogen, D-dimer and CRP. * $p<0.05$, ** $p<0.01$ and *** $p<0.001$.

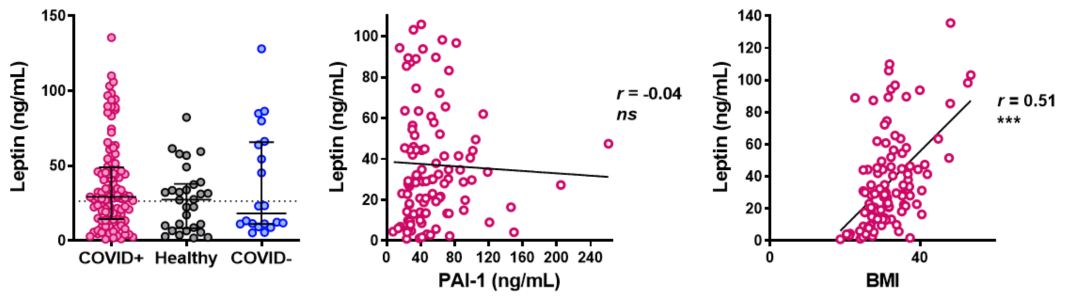

Supplementary Figure 5 – Leptin plasma levels do not differ between COVID-19 positive and negative patients and does not correlate with PAI-1 levels. (A) The concentration of leptin (ng/mL) was measured in plasma from patients with COVID-19 (COVID+), other non-COVID-19 respiratory infections (COVID-) and healthy controls by Simple Plex™ assays on Ella™. Dotted lines indicate antigen concentrations in pooled normal plasma. Data are shown as median ± interquartile range (IQR). Correlations of leptin with (B) PAI-1 and (C) BMI. *** p<0.001, ns = non-significant.

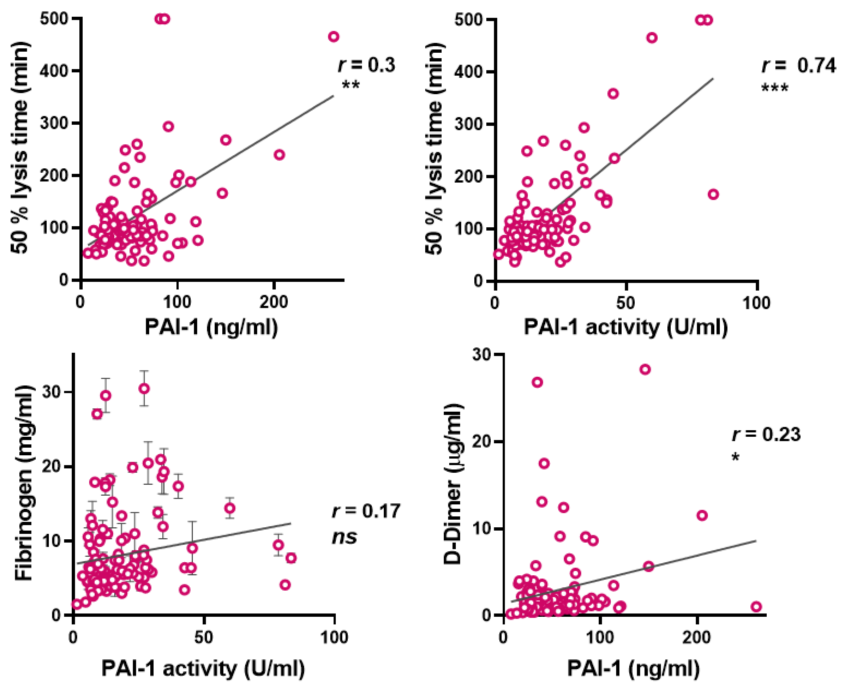

Supplementary Figure 6 – PAI-1 correlates with fibrinolytic activity
Pearson correlation coefficients (r) were calculated between PAI-1 antigen or activity with tPA -mediated lysis and with fibrinogen and D-dimer and PAI-1 activity. * p<0.05, ** p<0.01 and *** p<0.001.

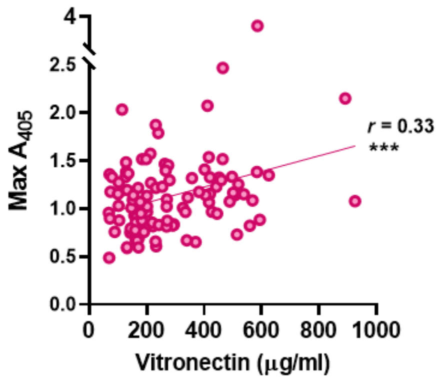

Supplementary Figure 7 – Vitronectin levels correlate with plasma clot maximum absorbance. Pearson correlation coefficients (r) were calculated between vitronectin and plasma clot maximum absorbance at 405nm. *** p<0.001.

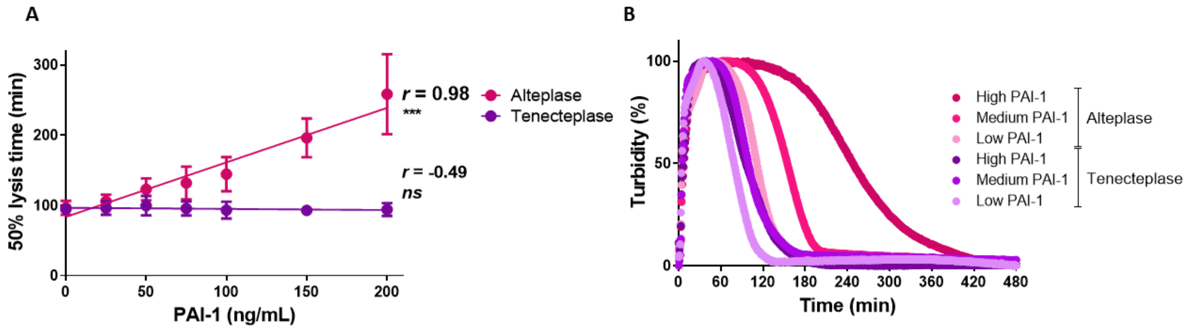

Supplementary Figure 8 –PAI-1 delays clot lysis by Alteplase but not Tenecteplase. Plasma clots (30 %) were formed in the presence of phospholipids (16 mM) ± Alteplase or Tenecteplase (300 pM). Clotting was initiated with CaCl2 (10.6 mM) and thrombin (0.1 U/ml) and absorbance readings at 405 nm were taken every min for 8 h at 37 °C. (A) Purified PAI-1 was spiked into pooled plasma at 0, 25, 50, 75, 100, 150 and 200 ng/mL. Data show the median ± IQR time to 50 % lysis of plasma clots (n=4). The relationship between 50% lysis time and PAI-1 concentration was significantly different between the two plasminogen activators, Alteplase and Tenecteplase. *** p<0.001. (B) Normalised clot lysis profiles of representative COVID-19 plasma with low <50 ng/mL [13.4-46.4 ng/mL]; medium 50-100 ng/mL [51.5 - 92.3 ng/mL] or high >100 ng/mL [103.3 - 260 ng/mL]) PAI-1 antigen (n=3 representative samples per PAI-1 grouping).
